# Boosted Hydrogen Evolution Catalysis Using Biomass-Derived Mesoporous Carbon Nanosponges

**DOI:** 10.3390/ijms26178502

**Published:** 2025-09-01

**Authors:** Sankar Sekar, Sutha Sadhasivam, Atsaya Shanmugam, Saravanan Sekar, Youngmin Lee, Sejoon Lee

**Affiliations:** 1 Division of System Semiconductor, Dongguk University, Seoul 04620, Republic of Korea; sanssekar@dongguk.edu (S.S.); atsyshanmu@dgu.ac.kr (A.S.); 2 Quantum-Functional Semiconductor Research Center, Dongguk University, Seoul 04620, Republic of Korea; 3 Department of Chemistry, CMS College of Engineering, Ernapuram, Namakkal 637003, Tamil Nadu, India; suthaasridhar@gmail.com; 4 Department of Mechanical Engineering, K. Ramakrishnan College of Technology, Trichy 621112, Tamil Nadu, India; nanosaran007@gmail.com

**Keywords:** biomass, neem leaves, ginkgo leaves, activated carbon, electrocatalysts, hydrogen evolution reaction

## Abstract

Carbon-based metal-free catalysts, particularly those such as biomass-derived mesoporous activated carbon (AC) nanostructures, hold great promises for cost-effective and sustainable electrocatalysis for enhancing hydrogen evolution reaction (HER) performance in green energy technology. Neem and ginkgo leaves are rich in bioactive compounds and self-doping heteroatoms with naturally porous structures and act as a low-cost, sustainable biomass precursors for high-performance HER catalysts. In this study, mesoporous AC nanoflakes and nanosponges were synthesized using biomass precursors of neem and ginkgo leaves through a KOH activation process. Notably, AC nanosponges derived from ginkgo leaves exhibited outstanding physicochemical characteristics, including a sponge-like porous morphology with a large specific surface area of 1025 m^2^/g. For electrochemical evaluation in 0.5 M H_2_SO_4_, the G-AC sample revealed superior electrocatalytic HER performance, with a remarkably low overpotential of 26 mV at −10 mA/cm^2^, a small Tafel slope of 24 mV/dec, and long-term durability over 30 h. These results depict biomass-derived mesoporous AC nanosponges to hold substantial potential for highly efficient hydrogen production, contributing significantly to the advancement of eco-friendly energy solutions.

## 1. Introduction

The urgent need for renewable energy sources is critical in addressing the escalating energy crisis and associated environmental challenges [1,2,3,4,5,6,7,8]. Green hydrogen has attracted considerable attention as a promising alternative energy source due to its environmental sustainability, high energy density, and zero carbon emissions [9,10,11]. Among various hydrogen production methods, electrocatalytic water splitting is regarded as the most effective, environmentally benign, and scalable approach for generating green hydrogen via the hydrogen evolution reaction (HER) [12,13,14,15]. As a key half-reaction in the water electrolysis process, HER offers a practical and cost-effective route for large-scale hydrogen production, making it a viable solution to both energy demands and environmental concerns [16,17,18]. However, the sluggish reaction kinetics and substantial overpotential associated with HER substantially limit the catalytic efficiency of water electrolysis systems [19,20,21,22]. Although platinum (Pt)-based materials persist among the highly efficient HER catalysts, their high cost and limited availability severely constrain their large-scale application [23,24]. Consequently, the development of low-cost, metal-free, acid-stable, and highly active HER catalysts derived from abundant natural resources is essential for advancing sustainable hydrogen production technologies.

Recently, carbonaceous materials and their nanostructures have attracted significant attention as HER electrocatalysts owing to their excellent electronic conductivity, chemical stability, and capacity to host electrochemically active species [25,26,27]. Among these, biomass-derived activated carbon (AC) has emerged as a promising candidate owing to its natural abundance, low cost, high conductivity, large surface area, tunable porous structure, and excellent durability [28,29,30,31]. As a result, substantial efforts have been devoted to synthesizing carbon nanomaterials from abundant biomass sources and waste materials to develop efficient carbon-based catalysts for energy conversion applications [32,33,34]. For example, Han et al. [35] prepared N-doped porous carbon fibers from natural cattail fiber, attaining a low overpotential of 244 mV and a Tafel slope of 70 mV/dec. Prabu et al. [36] synthesized hierarchical porous carbon from palm plants, recording an overpotential of 330 mV and a Tafel slope of 63 mV/dec. Similarly, Cao et al. [37] developed nitrogen-doped porous carbon from bean sprouts using the SiO_2_ template technique, obtaining an overpotential of 413 mV and a Tafel slope of 98 mV/dec. Additionally, Prabu et al. [38] derived nanoporous activated carbon sheets from Ooty Varkey food waste, demonstrating excellent HER activity with a low overpotential of 380 mV at 10 mA/cm^2^. Arul Saravanan et al. [39] prepared AC nanosheets from peanut shells via KOH activation, demonstrating outstanding HER performance with a notably low overpotential of 80 mV at 10 mA/cm^2^ and a Tafel slope of 75 mV/dec. Hoang et al. [40] synthesized Ni- and P-doped carbon from carrots using a facile one-step pyrolysis method, achieving an HER overpotential of 939 mV with a Tafel slope of 273 mV/dec. Among various biomass resources, neem leaves and ginkgo leaves stand out as promising sustainable precursors for producing AC nanostructures due to their natural richness, low cost, high carbon content, and eco-friendliness [41,42,43,44,45,46]. Utilizing these biomass sources offers an effective route for fabricating high-performance AC nanostructures. Rich in bioactive compounds (i.e., cellulose, protein, carbohydrates, calcium, vitamin, carotene, and phosphorous), self-doping heteroatoms (i.e., O, N, S) characteristics, high-reaction-active sites, unique leaf structures, and huge availability, and the fact that they are characterized by high surface areas and large porous natures, means that neem and ginkgo leaves are particularly well-suited for the fabrication of cost-effective biomass catalysts with high HER performance, and thus provide an environmentally sustainable platform for efficient electrocatalyst development. Despite these advantages, no studies to date have reported the use of neem leaf- and ginkgo leaf-derived mesoporous AC (N-AC and G-AC) nanostructures specifically as HER electrocatalysts.

Motivated by the aforementioned background, we synthesized mesoporous N-AC nanoflakes and G-AC nanosponges via the KOH activation method and evaluated their HER performances. Notably, the G-AC catalyst exhibited an impressively lower overpotential of 26 mV and a small Tafel slope of 24 mV/dec at 10 mA/cm^2^ in 0.5 M H_2_SO_4_. This work presents a comprehensive examination of the catalyst synthesis, material characterization, and electrocatalytic HER activities of the fabricated N-AC nanoflakes and G-AC nanosponges.

## 2. Results and Discussion

**Figure 1:**
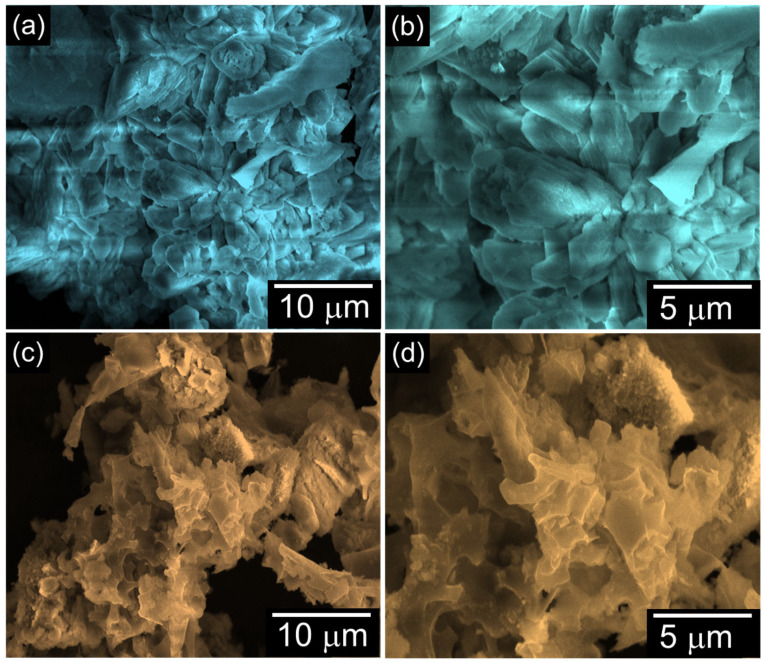
Low- and high-magnification FE-SEM images of (**a**,**b**) N-AC and (**c**,**d**) G-AC nanostructures.

Figure 1 displays the surface morphology of the N-AC and G-AC nanostructures derived from biomass neem leaves and ginkgo leaves, respectively, via the KOH activation method. The N-AC sample exhibits an irregularly stacked and aggregated layered flake-like structure (Figure 1a,b), while the G-AC sample displays an irregular, three-dimensional porous network with a sponge-like morphology (Figure 1c,d). Compared to N-AC, the G-AC structure offers greater porosity, a higher surface area, and improved accessibility to active sites, all of which are advantageous for enhancing HER activity.

**Figure 2:**
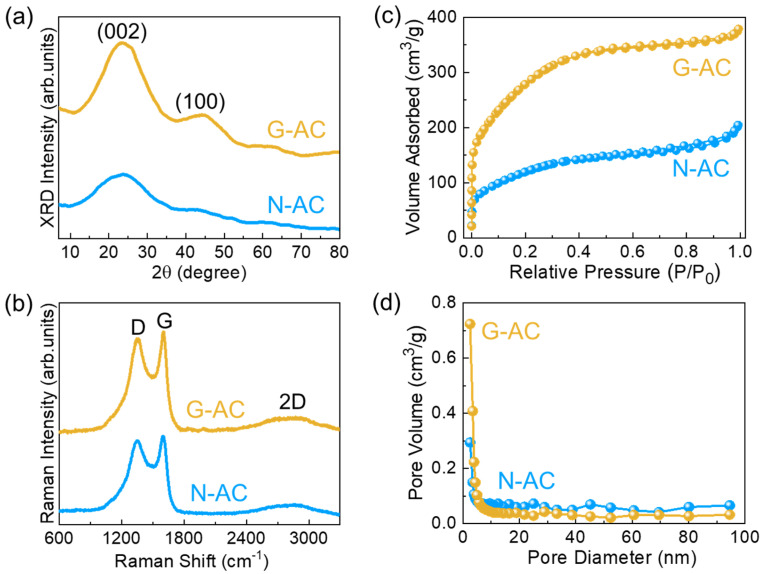
(**a**) XRD patterns, (**b**) Raman spectra, (**c**) N_2_-absorption and desorption isotherms, and (**d**) pore size distributions of the N-AC and G-AC nanostructures.

The structural properties of the N-AC and G-AC nanostructures were analyzed using XRD. Figure 2a shows the XRD patterns of both samples, which reveal two characteristic diffraction peaks at 23.6° and 44.3°, corresponding to the (002) and (100) planes of disordered carbon, respectively, indicating their amorphous nature [38,47,48]. Compared to N-AC, the G-AC sample exhibits a more intense (002) peak, suggesting a lower degree of structural disorder and a higher pore density [49,50]. This improved graphitization contributes to enhanced electrical conductivity in the G-AC nanostructure. No secondary phases were detected, confirming the high purity of both samples. The degree of graphitization and structural disorder in the carbonaceous materials was further examined via Raman spectroscopy. Figure 2b presents the Raman spectra of N-AC and G-AC, both of which show three prominent peaks at 1351 cm^−1^ (D band), 1603 cm^−1^ (G band), and 2853 cm^−1^ (2D band), indicating the graphitic characteristics of the synthesized carbon materials [13,31,35,51]. The D band arises from the A_1g_ vibrational mode, associated with structural defects and disordered vibrations at the edges of graphitic domains [52]. The G band corresponds to the E_2g_ mode of sp^2^-hybridized carbon atoms, characteristic of an ordered graphitic lattice [53]. The presence of the 2D band serves as a distinguishing feature of activated carbon [54]. The intensity ratios of the D and G bands (*I*_D_/*I*_G_) were calculated as 0.97 for N-AC and 0.94 for G-AC, indicating that G-AC has a lower degree of disorder and a higher level of graphitization compared to N-AC.

The specific surface area and porosity of the N-AC and G-AC nanostructures were analyzed using the BET and BJH techniques. Figure 2c shows the N_2_ adsorption–desorption isotherm curves for both samples, which exhibit Type IV physisorption isotherms with Type H4 hysteresis loops (as classified by IUPAC), confirming their mesoporous nature [36,48,55,56]. BET analysis exhibited surface areas of 433 m^2^/g for N-AC and 1025 m^2^/g for G-AC. As illustrated in Figure 2d, the pore surface areas were determined to be 164 m^2^/g for N-AC and 360 m^2^/g for G-AC. Furthermore, G-AC exhibited a higher total pore volume (0.5805 cm^3^/g) compared to N-AC (0.3122 cm^3^/g). The average pore sizes were determined to be 2.88 nm for N-AC and 2.26 nm for G-AC. The smaller average pore size, along with the significantly higher surface area and pore volume of G-AC, clearly indicates its superior mesoporous structure, which is critical for enhancing electrocatalytic HER efficiency.

**Figure 3:**
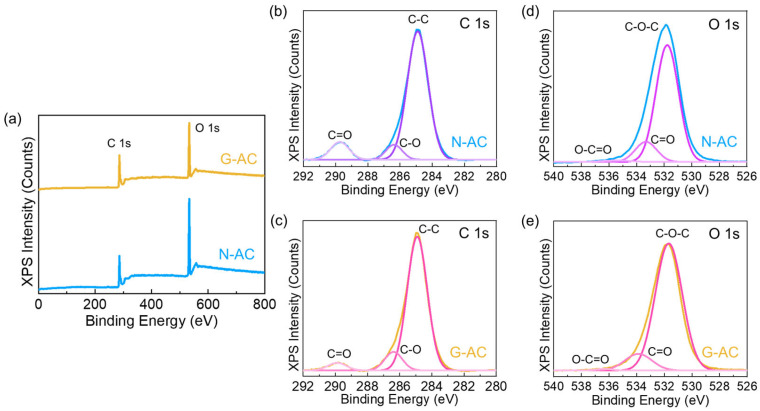
(**a**) Full survey XPS spectra of N-AC and G-AC nanostructures. (**b**) C 1s and (**c**) O 1s core-level spectra of N-AC nanoflakes. (**d**) C 1s and (**e**) O 1s core-level spectra of G-AC nanosponges.

The chemical bonding structures of the N-AC and G-AC were investigated by XPS measurements. The XPS full survey spectra of the N-AC and G-AC clearly reveal the presence of their constituent elements such as C and O (Figure 3a). The C1s core-level spectra of both samples (Figure 3b,c) exhibited three characteristic peaks at 284.8, 286.3, and 289.5 eV, corresponding to the C–C (sp^2^-hybridized domains), C–O (epoxy, hydroxyl), and C=O (carboxyl) functional groups, respectively [57,58]. Similarly, the O1s core-level spectra of the both samples (Figure 3d,e) also displayed three prominent peaks at 531.6, 533.8, and 536.2 eV, ascribed to the C–O–C, C=O, and O–C=O functional groups, respectively [59,60]. The abundance of these oxygen-containing functional groups can significantly alter the surface polarity of the carbon material, thereby enhancing the wettability at the electrode–electrolyte interface. This improved interfacial interaction is expected to facilitate more efficient ion transport, ultimately contributing to enhanced electrochemical performance of the catalyst.

**Figure 4:**
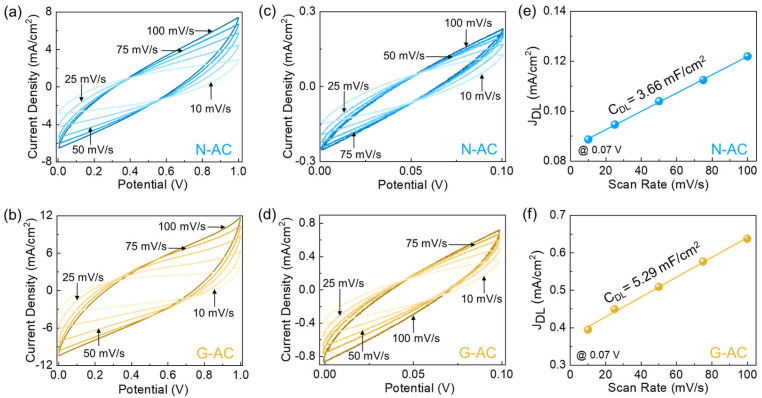
CV curves of (**a**) N-AC and (**b**) G-AC catalysts. Non-Faradaic CV curves of the (**c**) N-AC and (**d**) G-AC catalysts. Non-Faradaic *J*_DL_ at 0.07 V as a function of scan rate for (**e**) N-AC and (**f**) G-AC catalysts.

After confirming the structural characteristics of the fabricated catalysts, the electrocatalytic HER performance of N-AC and G-AC was systematically investigated. Figure 4a,b present the CV curves of the N-AC and G-AC catalysts recorded at scan rates ranging from 10 to 100 mV/s. Both catalysts exhibited typical rectangular-shaped CV profiles, indicating efficient electrical double-layer capacitive behavior [13,37,61,62]. As the scan rate amplified, the current density also rose, suggesting small diffusion resistance of the active materials. Notably, G-AC displayed a larger CV profile area and a higher current response compared to N-AC, implying a larger number of accessible active sites. The electrocatalytic HER activity was closely associated with the *C_dl_* and *ECSA* of the catalysts. The *C_dl_* values for N-AC and G-AC were determined to be 3.66 and 5.29 mF/cm^2^, respectively, from the non-Faradaic CV region at 0.07 V (see Figure 4c–f). Using Equations (9) and (10), the corresponding *ECSA* values were calculated as 105 cm^2^ for N-AC and 151 cm^2^ for G-AC. The significantly higher *C_dl_* and *ECSA* values of the G-AC catalyst indicate a larger number of exposed active sites and enhanced electrical conductivity compared to the N-AC catalyst.

**Figure 5:**
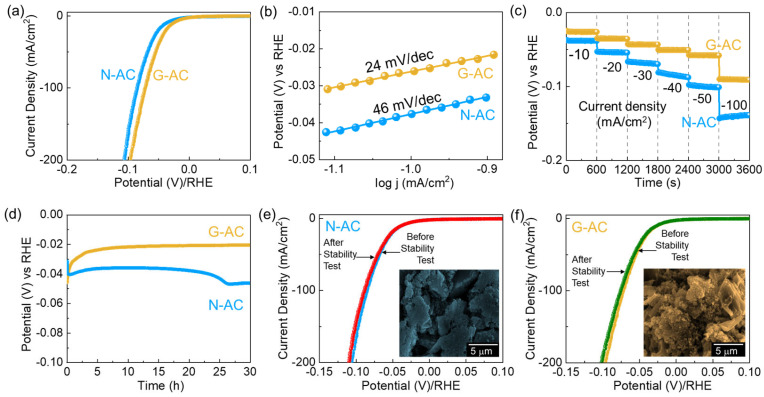
Electrocatalytic HER performance of N-AC and G-AC catalysts: (**a**) *i_R_*-corrected LSV curves, (**b**) Tafel plots, (**c**) chronopotentiometric profiles at various current densities (−10 to −100 mA/cm^2^), and (**d**) long-term durability analysis. LSV curves of the (**e**) N-AC and (**f**) G-AC catalysts before and after the HER durability test. FE-SEM images of the N-AC (inset of (**e**)) and the G-AC (inset of (**f**)) catalysts after the stability test.

The HER performance of the N-AC and G-AC catalysts was evaluated using LSV at a scan rate of 5 mV/s in 0.5 M H_2_SO_4_. Figure 5a presents the *i_R_*-corrected and without-*i_R_*-correction LSV curves (Figure S1) of the N-AC and G-AC catalysts. The *η* at a current density of −10 mA/cm^2^ was determined to be 40 mV for N-AC and 26 mV for G-AC, as calculated using Equations (11) and (12). The lower *η* value of G-AC is attributed to its enhanced electrical conductivity and greater number of catalytically active sites (i.e., higher *ECSA*) compared to N-AC. Figure 5b displays the Tafel curves of the N-AC and G-AC catalysts. The *S*_T_ values were determined to be 46 mV/dec for N-AC and 24 mV/dec for G-AC using Equation (13). The lower *S*_T_ value of G-AC indicates more favorable and efficient HER kinetics, consistent with the Volmer–Heyrovsky mechanism. Consequently, it is extensively accepted that the HER mechanism on the cathode surface proceeds through a sequence of electrochemical steps. In an acidic medium, this multistep process typically follows the reactions outlined below [63,64,65]:

(1) $$H_{3}O^{+}\;+\;M\;+\;e^{-}\to M\;-\;H\;+\;H_{2}O\;(Volumer)$$

(2) $$M\;-\;H\;+\;H_{3}O^{+}\;+\;e^{-}\to H_{2}\;+\;H_{2}O\;+\;M\;(Heyrovsky)$$

(3) $$2M\;-\;H\to 2M\;+\;H_{2}\;(Tafel)$$

where M denotes a vacant active site on the catalyst surface, while M–H refers to an adsorbed hydrogen species. The strength of the M–H bond plays a critical role in determining the HER kinetics of catalyst materials. In acidic solutions, the Volmer reaction (Equation (1)) involves an initial discharge of the hydronium ion and the formation of hydrogen intermediates (i.e., M-H), and the subsequent formation of H_2_ involves the electrochemical Heyrovsky step (Equation (2)) and the chemical Tafel step (Equation (3)). Therefore, the Volmer–Heyrovsky pathway is generally more efficient and favorable for HER activity. Compared to other electrocatalysts, the G-AC catalyst demonstrated superior HER performance, evidenced by lower *η* and *S*_T_ values, due to its surface oxygen groups, porous nature, larger *ECSA*, and improved electrical conductivity that enhanced the wettability, defect density, and charge transfer of the G-AC (Table 1).

The excellent HER performance of the N-AC and G-AC catalysts was further validated through CP measurements. As shown in Figure 5c, the G-AC catalyst consistently exhibited lower overpotentials at all applied current densities (−10, −20, −30, −40, −50, and −100 mA/cm^2^) than N-AC, confirming its higher catalytic efficiency. Figure 5d presents the long-term HER stability test of both catalysts at −10 mA/cm^2^ over 30 h. The G-AC catalyst exhibited excellent stability throughout the test duration, which can be attributed to its higher ECSA, greater porosity, and lower internal resistance. Furthermore, the LSV curves before and after the long-term stability test showed negligible deviation (Figure 5e,f), indicating microstructural and electrochemical robustness. These findings designate G-AC as a highly stable and efficient electrocatalyst for HER. Following the HER stability test, FE-SEM analysis was conducted to assess any microstructural changes in the catalysts. The N-AC catalyst displayed an aggregated flake-like structure (inset of Figure 5e), whereas the G-AC catalyst retained its original agglomerated sponge-like morphology (inset of Figure 5f). Furthermore, Raman measurements (Figure S2) showed that both catalysts retained their original features, indicating the high stability of the materials.

**Figure 6:**
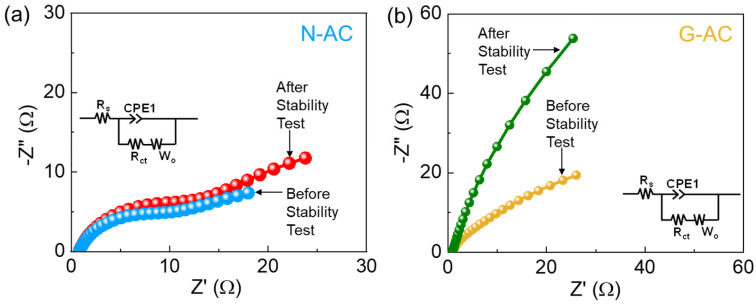
Nyquist plots of (**a**) N-AC and (**b**) G-AC catalysts before and after the stability test (inset: equivalent circuit model).

To further investigate the enhanced HER kinetics of the G-AC catalyst, EIS analysis was conducted. Figure 6 displays the Nyquist plots of the N-AC and G-AC catalysts, along with the corresponding equivalent circuit (inset). Both catalysts exhibited a linear region at low frequencies, indicating effective ion diffusion across the catalyst surface [13,39,71]. The absence of a semicircular region in both cases suggests high ionic diffusivity and excellent electronic conductivity [66,67,72]. The series resistance (*R*_s_) represents the combined resistance of the electrolyte, electrode, and interfaces, and a lower *R*_s_ indicates easier charge transport and reduced energy loss, leading to improve HER performance. Using the equivalent circuit model, the *R*_s_ values were calculated to be 0.94 Ω for N-AC and 0.83 Ω for G-AC. The lower *R*_s_ value and steeper Nyquist slope of the G-AC catalyst are attributed to its higher density of active sites, greater porosity, enhanced ion transport, and improved electrical conductivity. However, the post-stability EIS results show lower charge transfer resistance, confirming the catalyst’s long-term stability due to surface restructuring and improved electrode–electrolyte interaction. These results highlight the strong potential of the biomass-derived G-AC nanostructures from ginkgo leaves as sustainable and efficient HER electrocatalysts for future green hydrogen production.

## 3. Materials and Methods

### 3.1. Materials

All chemicals were purchased from Sigma-Aldrich (Seoul, Republic of Korea) and used without further purification. Neem leaves (*Azadirachta indica*) were collected from Tholudur, Tamil Nadu, India, and ginkgo leaves (*Ginkgo biloba*) were obtained from Seoul, Republic of Korea.

### 3.2. Preparation of Activated Carbon Nanostructures

**Figure 7:**
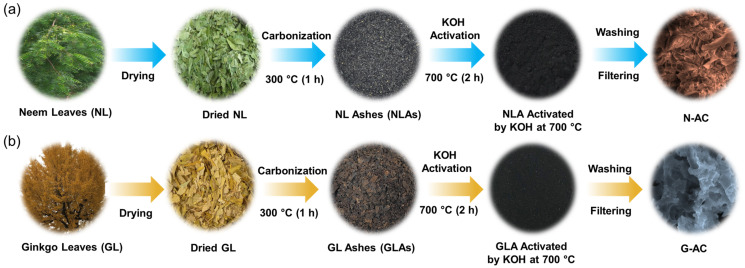
Schematic representation of the synthesis process of (**a**) N-AC and (**b**) G-AC nanostructures from neem and ginkgo leaves, respectively, via KOH activation.

Figure 7 presents a schematic illustration of the fabrication process for N-AC nanoflakes (Figure 7a) and G-AC (Figure 7b) nanosponges derived from biomass neem leaves and ginkgo leaves, respectively. Mesoporous AC nanoflakes and nanosponges were synthesized from these two biomass sources via the KOH activation method. Initially, the raw leaves were separated, thoroughly washed with deionized (DI) water to remove surface impurities, and sun-dried for seven days. The dried neem leaves (NL) and ginkgo leaves (GL) were then carbonized at 300 °C in an air atmosphere for 1 h to obtain carbonized ashes, referred to as NLAs and GLAs, respectively. Following carbonization, 3 g of each carbonized ash was mixed with 12 g of KOH using a mortar and subsequently annealed at 700 °C in an air atmosphere for 2 h. During KOH activation, oxygen- and carbon-containing functional groups on the carbon precursor reacted with KOH, resulting in the formation of various carbonaceous species such as potassium carbonate (K_2_CO_3_) and carbon monoxide (CO). This high-temperature activation process can be represented by the following chemical reactions [13,73,74]:

(4) $$2C\left(NLAs\;\left(or\right)\;GLAs\right)\;+\;6KOH\to 3H_{2}\;+\;2K+2K_{2}CO_{3}$$

(5) $$K_{2}CO_{3}\to K_{2}O\;+\;CO_{2}$$

(6) $$CO_{2}\;+\;C\to 2CO$$

(7) $$K_{2}CO_{3}\;+\;2C\to 2K+3CO$$

(8) $$K_{2}O\;+\;C\to 2K\;+\;CO$$

After activation, the resulting colloidal suspension was collected and stirred with DI water for 8 h to eliminate unreacted potassium complexes. The resulting mixture was then filtered, rinsed with DI water, and dried at 120 °C for 10 h. The AC nanopowders derived from neem leaves and ginkgo leaves are denoted as N-AC and G-AC, respectively.

### 3.3. Material Characterizations

The microstructures of the fabricated N-AC and G-AC catalysts were examined using field-emission scanning electron microscopy (FE-SEM). Their amorphous nature and vibrational properties were characterized via X-ray diffraction (XRD) and Raman spectroscopy, respectively. Pore size distribution and textural characteristics were analyzed using the Brunauer–Emmett–Teller (BET) and Barrett–Joyner–Halenda (BJH) methods.

### 3.4. Electrocatalytic HER Measurements

The electrocatalytic HER performance of the N-AC and G-AC catalysts was evaluated using an electrochemical workstation configured in a standard three-electrode setup. For electrode preparation, either N-AC or G-AC was dispersed in N-methyl-2-pyrrolidone to form a homogeneous slurry, which was subsequently coated onto stainless-steel substrates (1 cm^2^) and dried at 160 °C for 6 h. A coiled Pt wire and a saturated calomel electrode (SCE) were employed as the counter and reference electrodes, respectively. All HER measurements—including cyclic voltammetry (CV), linear sweep voltammetry (LSV), electrochemical impedance spectroscopy (EIS), and chronopotentiometry (CP)—were conducted in 0.5 M H_2_SO_4_. LSV was performed within the potential range of 0.1 to −1.2 V at a scan rate of 5 mV/s. All LSV measurements were calculated with 100% i_R_ compensation to ensure accurate potential values. CV measurements were carried out at scan rates ranging from 10 to 100 mV/s within a 0–1.0 V potential window. HER rate performance was evaluated by CP at current densities of −10, −20, −30, −40, −50, and −100 mA/cm^2^ for 10 min each. EIS analysis was conducted over a frequency range of 1 Hz to 10 kHz. The electrochemical double-layer capacitance (*C*_dl_) and electrochemically active surface area (*ECSA*) of the catalysts were calculated from the non-Faradaic CV region using the following equations [75,76,77]:

(9) $$J_{DL}\;=\;C_{dl}\;\times \;v/A$$

(10) $$ECSA\;=\;C_{dl}/C_{e}$$

where *C*_dl_ is the non-Faradaic capacitance, *J*_DL_ is the non-Faradaic current density, *A* is the electrode area, *v* is the scan rate, and *C*_e_ is the specific capacitance of the electrolyte, taken as 0.035 mF/cm^2^ for 0.5 M H_2_SO_4_ [78]. The overpotential (*η*) and Tafel slope (*S*_T_) values for the N-AC and G-AC catalysts were determined using the subsequent equations [19,35,39,79]:

(11) $$E_{RHE}\;=\;E_{SCE}\;+\;0.059\cdot pH\;+\;E_{SCE}^{0}$$

(12) $$E_{RHE}\;=\;\eta$$

(13) $$\eta \;=\;S_{T}\log\left(J\right)\;+\;c$$

where *J* represents the current density, *E*_RHE_ is the potential vs. the reversible hydrogen electrode (RHE), *c* is a fitting constant, and $E_{SCE}^{0}$ signifies the typical potential of the SCE.

## 4. Conclusions

The mesoporous N-AC and G-AC nanostructures were successfully synthesized from two different biomass sources—neem and ginkgo leaves—via the KOH activation process. The N-AC sample exhibited a stacked and aggregated layered flake morphology, whereas the G-AC sample displayed a three-dimensional porous sponge-like morphology with an enhanced specific surface area. Owing to its high specific surface area, substantial mesoporosity, and superior graphitization, the G-AC catalyst achieved a low overpotential of 26 mV at −10 mA/cm^2^ in 0.5 M H_2_SO_4_. Furthermore, the G-AC catalyst exhibited a small Tafel slope of 24 mV/dec and demonstrated excellent long-term durability. These results specify that porous G-AC nanosponges are auspicious candidates for high-performance HER electrocatalysts in future green hydrogen production.

## Figures and Tables

**Table 1:** Comparison of HER performance between biomass-derived N-AC and G-AC nanostructures and previously reported carbon-based electrocatalysts.

Biomass Resources	Catalyst	Activation Source	*η* (mV)	S*_T_* (mV/dec)	Electrolyte	Ref.
Ginkgo Leaves	G-AC	KOH	26	24	0.5 M H_2_SO_4_	This Work
Neem Leaves	N-AC	KOH	40	46	0.5 M H_2_SO_4_	This Work
Bean Sprouts	BS-800	HF Etching	413	98	0.5 M H_2_SO_4_	[37]
Carrots	PC	Thermal Annealing	939	273	0.1 M KOH	[40]
Ooty Varkey	NACS	KOH	380	85	0.5 M H_2_SO_4_	[38]
Cattail Fiber	NPCF	KOH	244	135	0.5 M H_2_SO_4_	[35]
Eucalyptus Leaves	ELC-700	KOH	39	36	0.5 M H_2_SO_4_	[13]
Palm Waste	HPNS	KOH	330	63	0.5 M H_2_SO_4_	[36]
Human Hair	HH-AC-700	KOH	16	51	0.5 M H_2_SO_4_	[16]
Commercial AC	D-AC	NH_3_	334	66	0.5 M H_2_SO_4_	[66]
Broccoli Stems	NA9	KOH	184	164	1 M H_2_SO_4_	[67]
Peanut Shells	PSAC	KOH	80	75	0.5 M H_2_SO_4_	[39]
Golden Shower Pods	N-PC	Urea	179	98	1 M KOH	[68]
Rice Husk	RH-CG-600	KOH	33	67	0.5 M H_2_SO_4_	[19]
Camellia Japonica Flower	SA-Came	KOH	154	89	1 M KOH	[69]
Tea Waste	G-ACOH	KOH	349	128	0.5 M H_2_SO_4_	[70]

## Data Availability

The original contributions presented in this study are included in the article/Supplementary Materials. Further inquiries can be directed to the corresponding authors.

## Supplementary Materials

The following supporting information can be downloaded at: https://www.mdpi.com/article/10.3390/ijms26178502/s1.
