# Supplementary material for: Boosted Hydrogen Evolution Catalysis Using Biomass-Derived Mesoporous Carbon Nanosponges
Source: Int J Mol Sci. 2025 Sep 1;26(17):8502. doi: 10.3390/ijms26178502 (PMC12429316; doi:10.3390/ijms26178502)
Supplement: Supplementary file 1 [file ijms-26-08502-s001.zip › ijms-3799020-supplementary.pdf]

Supplementary Materials

Boosted Hydrogen Evolution Catalysis Using Biomass-Derived Mesoporous Carbon Nanosponges

Sankar Sekar [1,2], Sutha Sadhasivam [3], Atsaya Shanmugam [1], Saravanan Sekar [4], Youngmin Lee [1,2,*] and Sejoon Lee [1,2,*]

1 Division of System Semiconductor, Dongguk University, Seoul 04620, Republic of Korea; sanssekar@dongguk.edu (S.S.); atsyshanmu@dgu.ac.kr (A.S.)
2 Quantum-Functional Semiconductor Research Center, Dongguk University, Seoul 04620, Republic of Korea
3 Department of Chemistry, CMS College of Engineering, Ernapuram, Namakkal 637003, Tamil Nadu, India; suthaasridhar@gmail.com
4 Department of Mechanical Engineering, K. Ramakrishnan College of Technology, Trichy 621112, Tamil Nadu, India; nanosaran007@gmail.com
* Correspondence: ymlee@dongguk.edu (Y.L.); sejoon@dongguk.edu (S.L.)

■ Electrocatalytic Properties of N-AC and G-AC

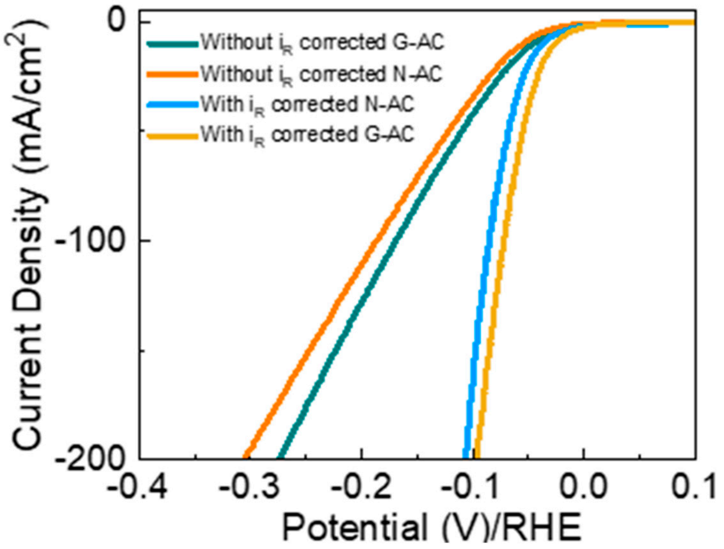

Figure S1: With and without i_R-corrected LSV curves of the N-AC and G-AC catalysts.

■ Structural Properties of N-AC and G-AC After Stability Test

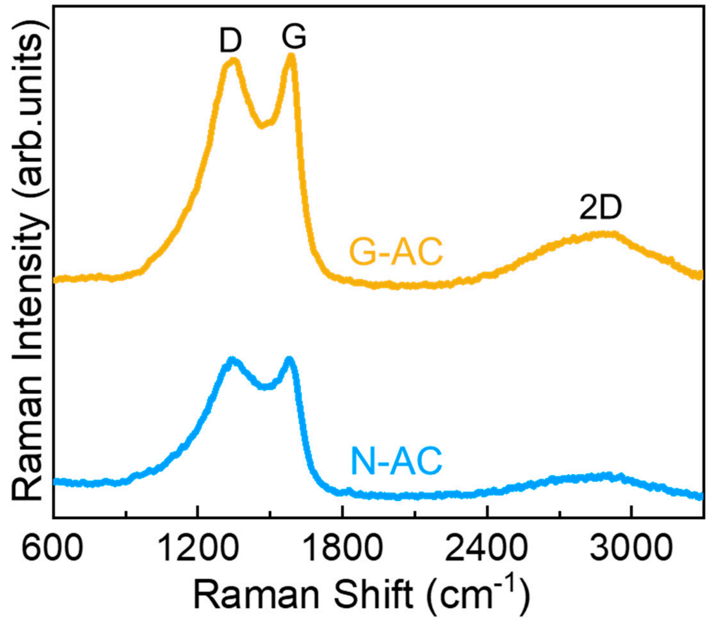

Figure S2: Raman spectra of the (a) N-AC and the (b) G-AC catalysts after the stability test.
